# Genome sequencing and genetic breeding of a bioethanol *Saccharomyces cerevisiae* strain YJS329

**DOI:** 10.1186/1471-2164-13-479

**Published:** 2012-09-15

**Authors:** Dao-Qiong Zheng, Pin-Mei Wang, Jie Chen, Ke Zhang, Tian-Zhe Liu, Xue-Chang Wu, Yu-Dong Li, Yu-Hua Zhao

**Affiliations:** 1 Institute of Microbiology, College of Life Sciences, Zhejiang University, Hangzhou, 310058, Zhejiang Province, P R China

**Keywords:** Bioethanol, *Saccharomyces cerevisiae*, Stress, Genome, RNA-Seq

## Abstract

**Background:**

Environmental stresses and inhibitors encountered by *Saccharomyces cerevisiae* strains are the main limiting factors in bioethanol fermentation. Strains with different genetic backgrounds usually show diverse stress tolerance responses. An understanding of the mechanisms underlying these phenotypic diversities within *S. cerevisiae* populations could guide the construction of strains with desired traits.

**Results:**

We explored the genetic characteristics of the bioethanol *S. cerevisiae* strain YJS329 and elucidated how genetic variations in its genome were correlated with specified traits compared to similar traits in the S288c-derived strain, BYZ1. Karyotypic electrophoresis combined with array-comparative genomic hybridization indicated that YJS329 was a diploid strain with a relatively constant genome as a result of the fewer Ty elements and lack of structural polymorphisms between homologous chromosomes that it contained. By comparing the sequence with the S288c genome, a total of 64,998 SNPs, 7,093 indels and 11 unique genes were identified in the genome of YJS329-derived haploid strain YJSH1 through whole-genome sequencing. Transcription comparison using RNA-Seq identified which of the differentially expressed genes were the main contributors to the phenotypic differences between YJS329 and BYZ1. By combining the results obtained from the genome sequences and the transcriptions, we predicted how the SNPs, indels and chromosomal copy number variations may affect the mRNA expression profiles and phenotypes of the yeast strains. Furthermore, some genetic breeding strategies to improve the adaptabilities of YJS329 were designed and experimentally verified.

**Conclusions:**

Through comparative functional genomic analysis, we have provided some insights into the mechanisms underlying the specific traits of the bioenthanol strain YJS329. The work reported here has not only enriched the available genetic resources of yeast but has also indicated how functional genomic studies can be used to improve genetic breeding in yeast.

## Background

Bioethanol is an important adjunct to fossil fuel because it is renewable, relatively environmentally innocuous, and compatible with the current fuel transport facilities. To date, bioethanol is mainly produced through the yeast-based fermentation of carbohydrates at about 33°C to give a final product concentration of 8–15% (v/v) [1,2]. Some novel processes, including high-gravity fermentation, high-temperature fermentation, and production from cellulose, intended to increase the economic and social benefits of ethanol, have been proposed and widely studied [2-6]. These processes, however, share the problem that they impose severe environmental stresses or inhibitors on yeast cells which greatly reduces their production efficiency. In addition, these stresses induce the formation of more by-products (mainly glycerol and acetic acid), consuming up to 5% of the carbon source [2-5].

The *Saccharomyces cerevisiae* strain S288c was, in 1996, the first eukaryotic genome to be sequenced [7]. In the 15 years that have passed since then, many functional genomic studies using the S288c genome as a reference sequence have greatly enriched our knowledge of how yeast cells respond to and resist various environmental stresses [8-16]. The information that has been produced cannot always be extrapolated to other yeast strains because of their diverse genomes and phenotypes [8,17,18]. Compared with laboratory strains, industrial strains generally show higher adaptability to specific environments; however, the genetic basis for their improved characteristics is not well understood. Comparisons of the genomes of strains with different backgrounds should help identify the sequence changes that play important roles in the tolerance of particular stresses. Because of the progress in genome sequencing technology, some industrial yeast strains, including AWRI1631, EC1118, JAY270, Vin13 and FostersO, have now been sequenced [19,20]. Comparisons of the publicly available *S. cerevisiae* genome sequences have revealed the clear signatures (single nucleotide polymorphisms (SNPs), insertions and deletions (indels), and novel ORFs) of different strains [18,20,21]. However, further studies are needed to explore how the genetic variations confer the specific phenotype of each strain. Of these industrial strains, JAY270 (PE-2 derived) which uses sugar cane as feedstock, is the only bioethanol strain [1]. Little is known about the genome structure and characteristics of other bioethanol strains.

In this study, we investigated the genetic characteristics of a bioethanol strain, YJS329, and the molecular mechanisms that underlie its phenotypic differences from the laboratory strain, BYZ1 (S288c-derived). YJS329 exceeded BYZ1 in fermentation rate and ethanol yield under different stress conditions, consistent with its greater tolerance of multiple stresses. Comparative genomic hybridization array and whole genome sequencing revealed many differences in the genomes of these two strains, including SNPs, indels, novel ORFs and changes in chromosome structure. Finally, we used RNA-Seq to determine how the genetic differences might affect the transcriptional profile and physiological metabolism of the two strains. Our study enriches the genetic resources for *S. cerevisiae* and deepens our knowledge of the effects of genetic variation on phenotypic diversity.

## Results

### Phenotypic and physiological characteristics of YJS329

**Figure 1:**
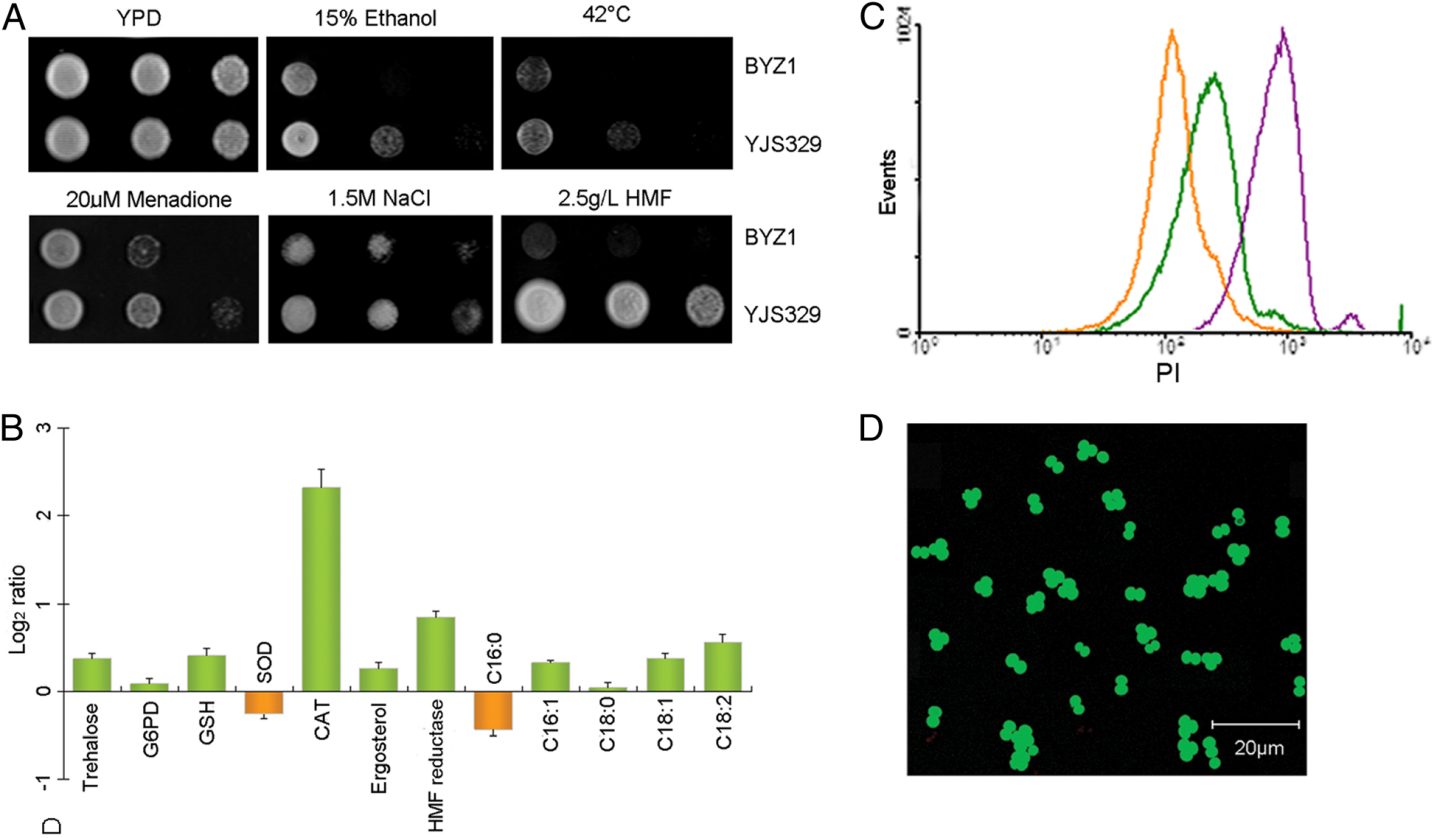
**Phenotypic and physiological traits of the bioethanol yeast strain YJS329.** (**A**) Growth of BYZ1 and YJS329 on plates with and without imposed stresses. Cells were grown in YPD liquid medium at 30°C for 20 h, and 3-μL 10-fold serial dilutions of each sample were spotted onto YPD plates. The YPD plates were then subjected to the indicated stressors. Three independent experiments were conducted, and typical data from one of them are shown. (**B**) Relative content of physiological and biochemical factors in YJS329. Cells were cultured in YPD for 18 h and then collected. Measurement of the trehalose, glucose-6-phosphate dehydrogenase (G6PD), glutathione (GSH), superoxide dismutase (SOD), catalase (CAT), ergosterol, hydroxymethylfurfural (HMF) reductase, palmitic acid (C16:0), palmitoleic acid (C16:1), oleic acids (C18:1), and linoleic acid (C18:2) content was then performed. The values are expressed as log2 ratios (YJS329/BYZ1) that represent the mean of three independent cultured samples (bars indicate SD). (**C**) Ploidy determination of YJS329 by flow cytometry. The stationary-phase cells of yeast strain BYZ1 (orange), YJS329 (green), and a triploid strain ZTW3 (violet) were fixed with 70% ethanol and stained with propidium iodide. DNA content corresponds to the intensity of red fluorescence. (**D**) Sporulation efficiency of YJS329. Cells were precultured in YPD and sporulated in sporulation medium. Asci were stained with fluorescein diacetate and then imaged with a confocal laser scanning microscope.

In comparisons of fermentation performance, YJS329 had a slightly higher fermentation rate than BYZ1 but they each produced similar amounts of ethanol in a 38-h period under standard conditions (Table 1 and Additional file 1). At higher temperatures and under higher gravity conditions, the ethanol yield of YJS329 was 16.6% and 12.1% (*t* test, *P* <0.001) higher than that of BYZ1, respectively (Table 1 and Additional file 1). In addition, under the three fermentation conditions tested, YJS329 produced more glycerol, whereas BYZ1 produced more acetic acid (Table 1). Consistent with the fermentation tests, YJS329 grew faster than BYZ1 when exposed to stress factors (ethanol, high temperature, osmotic stress, and oxidative stress; Figure 1A) and YJS329 also exceeded BYZ1 in tolerance to the furan derivative hydroxymethylfurfural (HMF), a major inhibitory compound in the fermentation of lignocellulosic hydrolysates.

**Table 1:** Performance of the yeast strains, BYZ1 and YJS329, under different fermentation conditions

**Fermentation conditions**	**Strain**	**Ethanol produced (g/L)**	**Glycerol produced (g/L)**	**Acetic acid produced (g/L)**	**Residual glucose (g/L)**	**Dry weight (g/L)**
Normal	BYZ1	71.89 ± 0.67	3.95 ± 0.08	1.06 ± 0.02	0.13 ± 0.00	9.4 ± 0.14
	YJS329	71.15 ± 0.72	4.36 ± 0.07^a^	0.44 ± 0.01^A^	0.03 ± 0.00	9.1 ± 1.02
High temperature	BYZ1	60.18 ± 0.53	3.68 ± 0.09	1.21 ± 0.02	25.07 ± 0.34	3.9 ± 0.11
	YJS329	70.16 ± 0.55^B^	4.90 ± 0.07^B^	0.78 ± 0.01^B^	1.11 ± 0.04^B^	3.5 ± 0.09^b^
High gravity	BYZ1	111.58 ± 1.19	5.93 ± 0.04	1.50 ± 0.04	21.45 ± 0.25	9.2 ± 0.13
	YJS329	125.10 ±1.13^C^	9.04 ± 0.14^C^	0.61 ± 0.02^C^	1.24 ± 0.01^C^	8.8 ± 0.08^c^

^a-c^indicate a significant difference between YJS329 and BYZ1 under normal (letter a), high temperature (letter b), and high gravity (letter c) conditions at the 0.05 level using *t* test.

^A-C^indicate a significant difference between YJS329 and BYZ1 under normal (letter A), high temperature (letter B), and high gravity (letter C) conditions at the 0.01 level using *t* test.

We compared YJS329 and BYZ1 using some of the main anti-stress indicators, including trehalose accumulation, antioxidation factors, HMF reductase, and membrane compositions. YJS329 accumulated 1.29-fold ((*t* test, *P* <0.05) more intracellular trehalose, a nonspecific protectant that can maintain the function of macromolecules and membrane integrity under multiple stresses (Figure 1B) [22]. Consistent with its better menadione tolerance, YJS329 showed 1.32-fold (*t* test, *P* <0.05) higher glutathione content and 5-fold (*t* test, *P* <0.001) catalase (CAT) activity than BYZ1. In yeast cells, glutathione and CAT are important for the elimination of the reactive oxygen species that are caused by oxidizing agents or by other stresses [23]. HMF is formed as a result of hexose degradation during the process of lignocellulosic hydrolysis [24]. The chemical toxicity of HMF can be reduced by HMF reductase which converts the aldehyde functional group into an alcohol group in yeast cells [24,25]. Compared to BYZ1, the higher intracellular HMF reductase activity (*t* test, *P* <0.05; Figure 1B) of YJS329 might partly contribute to its increased resistance to HMF. The results in Figure 1B show that, of the various membrane compounds, more ergosterol, palmitoleic acid (C_16:1_), oleic acid (C_18:1_), and linoleic acid (C_18:2_) were detected in YJS329 (*t* test, *P* < 0.05). These findings indicated that there was significant variation in cellular components and physiological state between the YJS329 and BYZ1 strains.

### Genome structure of YJS329

**Figure 2:**
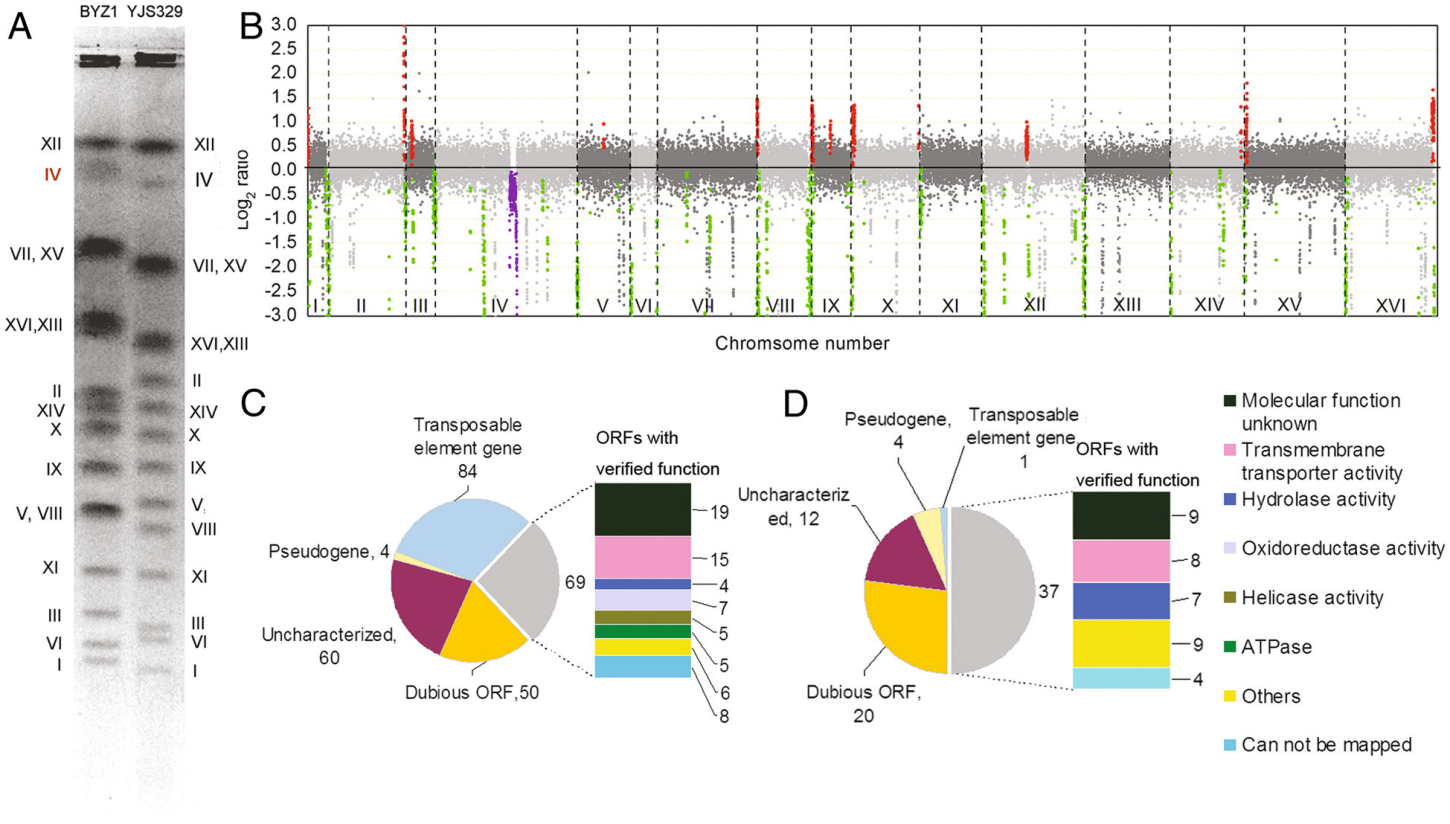
**Genome structure analysis of YJS329.** (**A**) Pulse-field gel electrophoresis of the BYZ1 and YJS329 chromosomes. (**B**) Comparison of the genome structures of BYZ1 and YJS329 by array-comparative genomic hybridization. Amplified regions and underrepresented regions in YJS329 are shown in red and green, respectively. The violet region represents the amplified regions of chromosome 4 in BYZ1. (**C**) Functional classification of the lost genes in YJS329. (**D**) Functional classification of the amplified genes in YJS329.

The DNA content of YJS329 was less than that of a triploid strain ZTW3 but close to that of BYZ1 (Figure 1C). After being grown in sporulation medium for 3–5 days, YJS329 showed an overall sporulation efficiency of 92%, producing mostly asci with two or three ascospores (Figure 1D). The pulse-field gel electrophoresis (PFGE) results revealed that YJS329 and BYZ1 differed distinctly in the length of their chromosomes; the exceptions were chromosomes 9, 10 and 14 (Figure 2A). The karyotype of YJS329 is more regular than the karyotypes of some other industrial strains [1,20], because the two homologs of each of the YJS329 chromosomes were the same length. The array-comparative genomic hybridization revealed that there were no big chromosomal aberrations in the genome of YJS329. The regions of the chromosomes that were underrepresented in the YJS329 genome (the green regions in Figure 2B) compared with in BYZ1 contain 267 ORFs (Figure 2C and Additional file 2). Most of these ORFs are located near the telomeres, long terminal repeat retrotransposons, or on tandemly repeated arrays. The regions of the chromosomes that were amplified in YJS329 relative to BYZ1 are shown in red in Figure 2B. Expressed products were identified for up to 50% of the ORFs in the amplified segments (Figure 2D and Additional file 2). The expressed genes include three hexose transport genes (*HXT8*, *HXT9*, and *HXT11*), four genes involved in maltose metabolism (*MAL12*, *MAL31*, *MAL32*. and *MAL33*), and four alpha-glucosidase genes (*IMA25*). The region of chromosome 4 that was amplified in BYZ1 (shown in purple in Figure 2B) led to the size differences between the homologs of chromosome 4 in this strain (Figure 2A). The RT-qPCR results confirmed that this amplification was present in the parent strain BY4742 before the generation of BYZ1 in the present work (See Additional file 3). This rearrangement was apparently Ty-derived as this region is flanked by the Ty elements YDR180W-A and YDRCTy1-3. Although the laboratory strains BY4741 and BY4742 have been used extensively in genetic research, this amplification has not been reported until now.

### Whole genome sequencing of YJS329

To investigate the genetic traits of YJS329, we isolated the haploid strain YJSH1 which, under certain conditions, is indistinguishable in ethanol yields from its parent strain YJS329 (See Additional file 4), for whole genome sequencing (See Additional file 5).

#### SNPs

**Figure 3:**
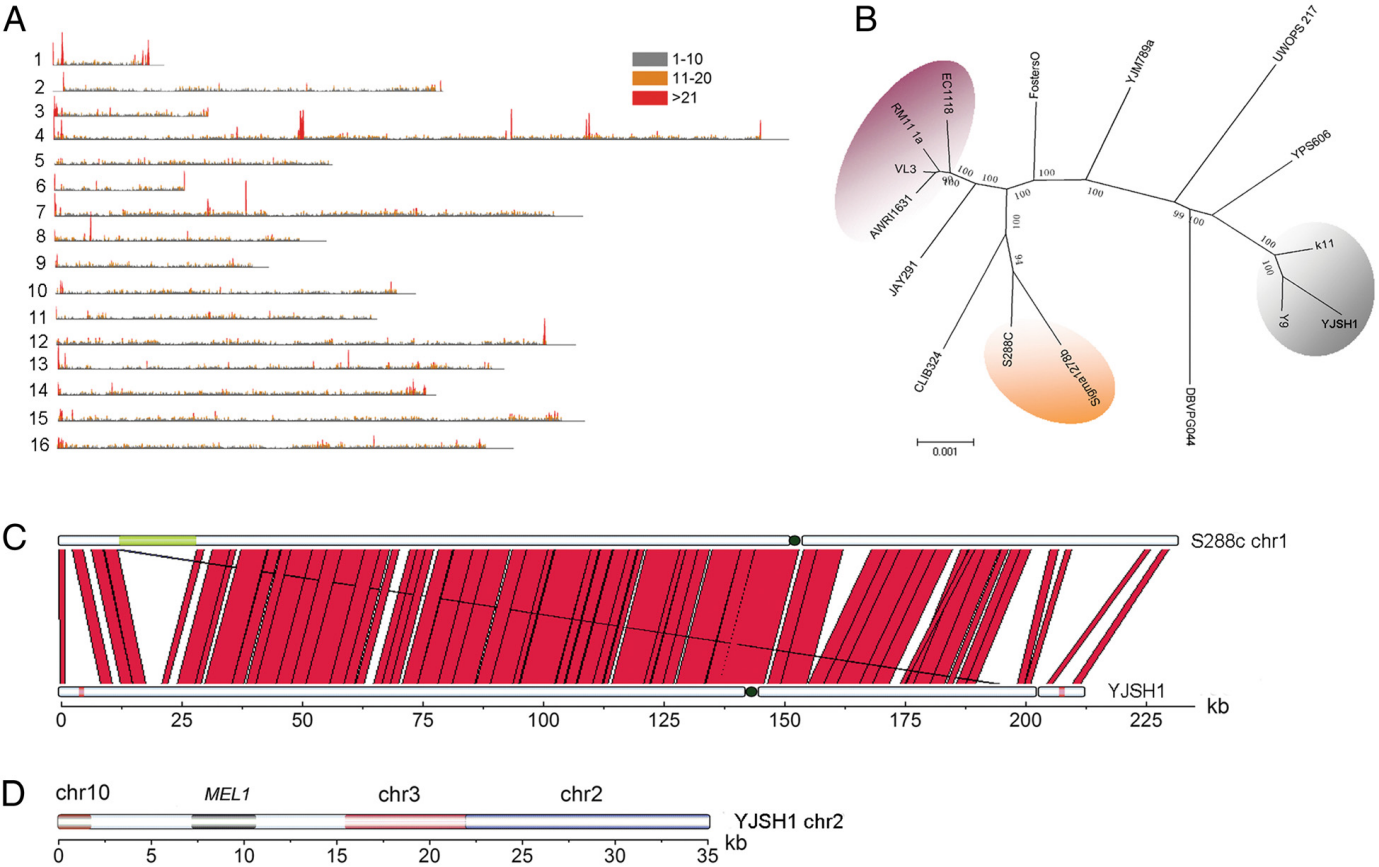
**Genome variation and genetic distance revealed by whole-genome sequencing.** (**A**) The distribution and density of SNPs in the YJSH1 genome within a sliding window of 1,000 bp. (**B**) A neighbor-joining tree representing the genetic distances between strains calculated from the total number of SNPs present in whole-genome alignments. The wine strains group is shown in plum, the laboratory strains in orange, and the sake strains in gray. (**C**) Chromosomal rearrangement events on chromosome 1 of the YJS329 genome. The full-length chromosome 1 sequences were aligned using the Artemis Comparative Tool (13). Sequences with >85% similarity are connected by red lines and sequences with <85% similarity or with no similarity are indicated by the white gaps. The green box indicates the largest indel on chromosome 1 of YJS329 and the red boxes indicate the novel ORFs *EPH1* (left) and *BIO6* (right). (**D**) From left to right, the sequence at the 5’ end of chromosome 2 in YJS329 was similar to regions of the sequences from chromosome 10 of S288c, gene *MEL1,* and chromosome 3 of S288c.

We identified 64,998 SNPs within the aligned regions of the YJSH1 and S288c genomes (the location of the SNPs and their annotations are listed in Additional file 6). The average SNP density was 5.73 per kilobase throughout the genome but the density was not constant across individual chromosomes (Additional file 5 and Figure 3A). A total of 39,098 SNPs were found in the ORFs and 38.7% of them resulted in non-synonymous mutations. We observed that genes (e.g. *HXT6*, *HXT7*, and *ARO3*) with redundant functions tended to accumulate more SNPs, which was consistent with their lower hybridization signals in the array-comparative genomic hybridization. Using the number of SNPs separating any two isolates as an estimation of their relatedness, we constructed a neighbor-joining tree that represented the genetic distances among 16 yeast strains. The tree shows that the bioethanol strains JAY291 and YJS329 displayed the closest evolutionary relatedness to the wine and sake strains, respectively (Figure 3B).

#### Indels

Based on the consensus YJSH1 genomic sequence, 412,794 bp that were absent in YJSH1 were identified in the S288c genome and 174,269 bp that were absent in S288c were identified in the YJSH1 genome (the location of the indels and their annotations were listed in Additional file 6). This analysis confirmed that some of the underrepresented regions in YJS329 genome (Figure 2B) were sequences that either were lost in this industrial strain or acquired in S288c. For example, the YJS329 genome had only one copy of *CUP1* and *ENA1*, and none of the *ASP3* genes found in S288c. We also identified 21 Ty elements in the YJS329 assembly (9 Ty1, 6 Ty2, 4 Ty3, 1 Ty4, and 1 Ty5), whereas 50 Ty elements have been identified in the S288c genome. The amplification of the Ty3 elements was consistent with the results of comparative genome hybridization for YJS329 (See Additional file 2).

#### ORFs

A total of 5,602 ORFs (common to S288c and excluding dubious ORFs) were predicted for the nuclear genome of YJS329 (the location of the ORFs and their annotations were listed in Additional file 7). Predictions indicated that 142 ORFs had in-frame stop codons, 129 ORF were affected by frame shifts, and 27 ORFs had lost start or stop codons because of the presence of SNPs or indels. For example, the *HO* gene of YJS329 had both an in-frame termination (the C at 238 bp was changed to T) and frame shift (the C at 413 bp was missing) (verified by PCR using YJS329 DNA as the template) that explained the heterothallic life cycle of YJS329. In addition, the YJS329 genome has some ORF sequences that were not present in S288c (Additional file 7); however, nearly all of these ORFs could be found in the genomes of other *S. cerevisiae* strains. One such example is the ORF *EPH1* that encodes the epoxide hydrolase (E.C. 3.3.2.3) that catalyzes the hydration of chemically reactive epoxides to their corresponding dihydrodiol products. A recent study suggested that *EPH1* in the *S. cerevisiae* genome was the result of an introgression event from S. *paradoxus* and the *S. paradoxus EPH1* gene may itself be a result of horizontal transfer from bacteria [26].

#### Structural variations

Compared to the strictly diploid *S. cerevisiae* S288c, many industrial yeast strains display chromosomal copy number variations (CNVs). Whole-chromosome amplifications had been observed in the AWRI796, VL3, FostersO and FostersB strains [20]. Although no large chromosomal aneuploidy or length polymorphisms were observed in the genome of YJSH1, some chromosomal rearrangement events in the YJSH1 genome were observed. The largest indel in the YJS329 genome was the 12.5-kb deletion in chromosome 1 region (11,872–24,331 bp; Figure 3C). The 5’ end of chromosome 2 in YJS329 was apparently subjected to constant remodeling (verified by PCR using YJS329 DNA as the template). In this region two elements from the S288c genome, chromosome 10 (729,223–727,336 bp) and chromosome 3 (315506–307348 bp), and a region that is absent in S288c genome (a BLASTN search showed that this region contained a *MEL1* gene that has been found in *S. carlsbergensis* and in other *S. cerevisiae* strains), were found in YJSH1 (1–23,308 bp; Figure 3D).

### Comparison of BYZ1 and YJS329 transcription using RNA-Seq

To investigate transcription differences at single-nucleotide resolution between BYZ1 and YJS329, poly(A)-enriched mRNAs from BYZ1 and YJS329 were used for high-throughput Illumina sequencing. Overall, 90.9% of the reads mapped to unique genomic regions; 81% mapped to known reference genes when 2-bp mismatches were allowed (Additional file 8). Compared to BYZ1, 888 of the YJS329 genes were up-regulated and 1,433 were down-regulated (*P* <0.001; Additional file 9). The functions of the up-regulated genes mainly fell within the oxidoreductase, peptidase activity and transporter-related processes categories (Additional file 10). For example, *SFA1* which is involved in the detoxification of formaldehyde and long-chain and complex alcohols formation [24,27] displayed more than a 15-fold increase in mRNA abundance in YJS329. The fair number of the up-expressed genes involved in transport processes in the YJS329 sample suggested that this strain might have higher adaptability to multiple nutrition shortages than BYZ1. The down-regulated genes were mainly involved in the functional categories of DNA/protein binding, ribosome biogenesis, and structural molecules (Additional file 10).

**Figure 4:**
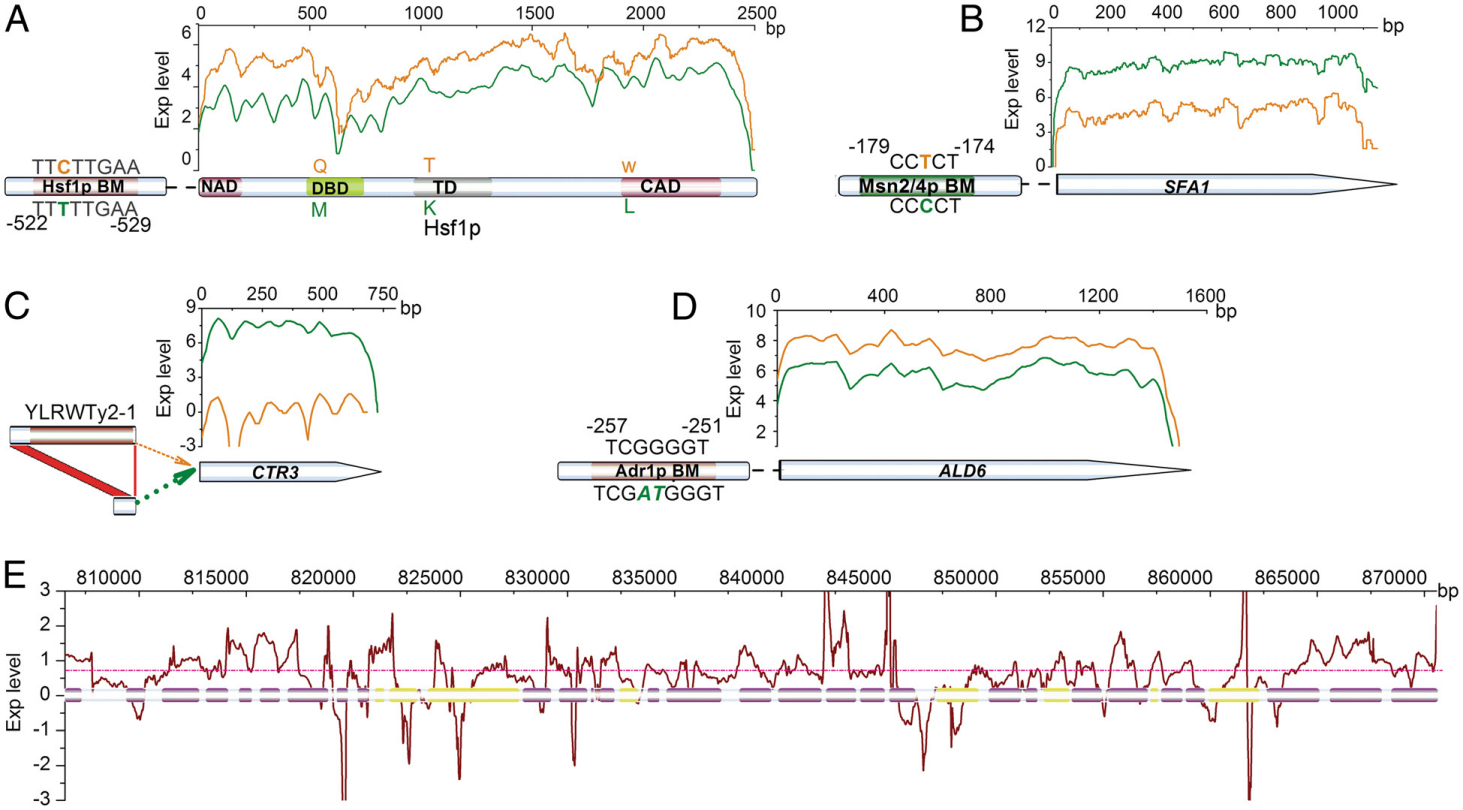
**The effects of genomic variations on the transcriptional differences between BYZ1 (orange) and YJS329 (green).** (**A**) Comparison of expression levels of *HSF1* in BYZ1 and YJS329 within a sliding window of 50 bp. The N-terminal activation domain (NAD), DNA-binding domain (DBD), trimerization domain (TD), and C-terminal activation domain (CAD) of the Hsf1p [33] are highlighted by colored boxes. The orange letters represent the corresponding amino acids in BYZ1; the olive letters represent those in YJS329. (**B**) Comparison of the promoter and the expression levels of the *SFA1* gene in BYZ1 and YJS329. The green box in the *SFA1* promoter represents the Msn2/4p binding motif in YJS329. (**C**) The insertion of a Ty2 element into the *CTR3* promoter greatly decreased the expression of the *CTR3* gene in BYZ1 (sliding window of 50 bp). (**D**) The down-regulation of *ALD6* in YJS329 might be caused by the loss of the Adr1p binding motif in the promoter (sliding window of 50 bp). (**E**) The relative expression level of the amplified region located on chromosome 4 of BYZ1, represented by the log2 ratio (BYZ1/YJS329), within a sliding window of 100 bp. The red dotted line indicates the mean value of the relative expression level. The up-regulated genes in the amplified region are indicated by violet boxes (*P* < 0.001); the genes that were not differentially expressed in this region are indicated by yellow boxes (*P* > 0.001).

Among these differentially expressed genes, we focused specifically on the transcriptional activity of the genes that are closely related to the anti-stress factors. Consistent with the analyses at the physiological and biochemical levels, the genes in ergosterol and fatty acid biosynthesis, and the genes encoding catalases were highly expressed but to different degrees in YJS329 (Additional file 11). We also found five transcription factors (*HAP1*, *MSN2/4*, *ARR1*, and *HSF1*) which are known to be major regulators that control critical cellular processes and response to environmental conditions [12,13,16], that displayed significantly different expression patterns in the two strains (Additional file 10). Transcription regulation network analyses (Additional file 10) revealed that a large proportion of the up-regulated genes (*TPS2* and *TSL1* in trehalose metabolism, *OLE1* and *ELO1* in oleic acid biosynthesis, and the catalase coding gene *CTT1*) was regulated by the Msn2/4p transcription factor, whose expression is itself dependent on or induced by other transcription factors (such as Hap1p) [12]. Although the zinc-finger transcription factor *HAP1* has a larger number of reads per kilobase of exon region per million mapped reads (RPKM) in BYZ1, the Hap1 protein is inactivated by a Ty1 insertion in the carboxy terminus [28]. The absence of this interrupting Ty1 element in the YJS329 protein may explain why the *HAP1*-regulated genes involved in the synthesis of fatty acids and ergosterol, such as *FAS1*, *FAS2*, *ERG2*, *ERG5*, *ERG11*, and *ERG25*, were expressed at a higher level in this strain (*t* test, *P <*0.001). Except for *ZIM17*, most of the genes that code for heat-shock proteins and the transcription factor Hsf1p showed less mRNA expression activity in YJS329 compared to BYZ1 (Additional file 10). An in-vitro experiment showed that the efficiency of the HSF1-promoter in YJS329 was 16% lower than in BYZ1 (*t* test, *P* <0.05; Additional file 12). Compared to BYZ1, a SNP in the *HSF1* promoter in YJS329 resulted in the loss of the Hsf1p binding motif which may be important for the variations in *HSF1* expression (Figure 4A). Furthermore, three amino acid substitutions in the functional domains of Hsf1p may impede its interaction with the promoters of heat shock proteins; however, this supposition needs further experimental verification.

As well as the destruction of binding motifs in transcription factors, SNPs can also create new binding motifs. The Msn2/4p and Cat8p binding sites in the promoter of *SFA1* from YJS329 are examples of new motifs that may strengthen the expression of the *SFA1* gene (Figure 4B and Additional file 12) which plays a role in the detoxification of furan derivatives [24]. Indels were also important contributors to transcription differentiation among the two strains. An obvious example in BYZ1 is the interruption of *CTR3* (which encodes a high-affinity copper transporter responsible for copper uptake when environmental copper is low [29]) by the insertion of a Ty2 element [30]. This insertion might explain the much lower expression activity of *CTR3* in BYZ1 compared to YJS329 (Figure 4C). Further, small indels in the trans-elements can directly modify mRNA expression and phenotypic traits in different strains. The down-regulated expression of *ALD6* in YJS329 (whether grown in YPD medium or under fermentation conditions and verified by RT-qPCR; *t* test, *P* <0.001), a major gene in acetic-acid formation, probably resulted partly from the insertion of two bases in the Adr1p binding motif in the *ALD6* promoter (Figure 4D and Additional file 12). In BYZ1, when the two copies of *ALD6* were deleted, the strain produced 56% less acetic acid and 17% more glycerol under the normal fermentation conditions (Additional file 13). This result indicated that the lower expression of *ALD6* in YJS329 could be one of the causes of the different patterns of by-product (acetic acid and glycerol) production in YJS329 and BYZ1. Chromosomal aneuploidy accompanied by CNVs in large DNA regions is a ubiquitous phenomenon in yeast populations [20,31]. As indicated in Figure 4E, the expression levels of regions with CNVs apparently dependent on gene dosage. The average read depth of the amplified region on chromosome 4 of BYZ1 was 1.59 times that in YJS329, close to the increased DNA dosage.

Using RNA-Seq, we detected the expression of the unique ORFs at the whole-transcription-profile level. Among these ORFs (Annotation details are in Additional file 7), *MEL1* had the highest RPKM; others, such as *YJM-GNAT*[32], showed minimal expression. Additional file 13 shows the expression level and boundary of the predicted ORF *chr06.orf003*, which provides further evidence of the existence of this novel ORF which is absent in other *S. cerevisiae* strains. RT-qPCR analyses revealed that the expression of some unique ORFs depended on the growth phase and other conditions (Additional file 14). When grown in YPD medium, all five of the selected genes (especially *BIO6*) showed the highest expression at the exponential phase. The ORFs *YJS-HE* and *MEL1* were significantly up-regulated under ethanol fermentation, whereas the others were down regulated, indicating the different psychological roles of these unique genes.

### Genetic breeding strategies for YJS329

**Figure 5:**
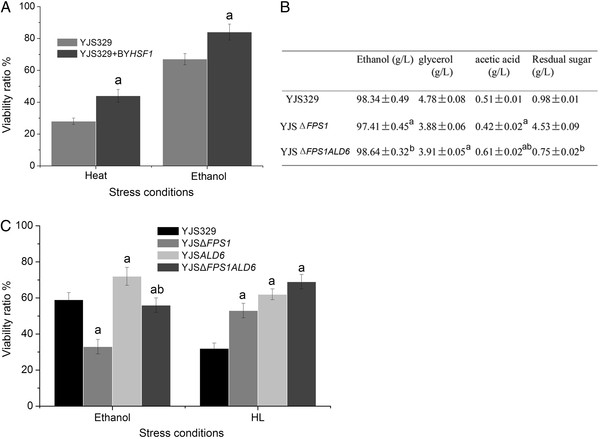
**Breeding strategies for YJS329.** (**A**) After heat and ethanol treatment, the moderate up-expression of *HSF1* in YJS329 improved its viability. Strains YJS329 and YJS329 + BY*HSF1* (the *HSF1* from BYZ1was expressed in YJS329) were pre-cultured in YPD medium and 1 mL cells (cell density was adjusted to OD_600_ = 1) were then subjected to either heat (55°C, 6 min) or ethanol (15% v/v in YPD liquid medium, 10 h) treatments. The “a” indicates a significant difference between YJS329 + BY*HSF1* and YJS329 (**B**) The impact of deletion of *FPS1* and overexpression of *ALD6* on the YJS329 fermentation process. The fermentation medium contained 220 g/L glucose, 10 g/L yeast extract, 20 g/L peptone. Data represent mean ± SD of three individual cultures. The “a” indicates a significant difference between YJS329Δ*FPS1* and YJS329; “b” indicates a significant difference between YJS329Δ*FPS1ALD6* and YJS329Δ*FPS1* using the *t* test at the 0.05 level. (**C**) Deletion of *FPS1* and overexpression of *ALD6* improved the viability ratio of after treatment with ethanol (15%, v/v) and lignocellulosic hydrolysate (LH, containing 4 g/L acetic acid, 1 g/L furfural, and 1 g/L 5-HMF, pH4.5) for 10 h. The “a” and “b” letters have the same meaning as in Figure 5B.

Hsf1p is a conserved transcription factor that regulates hundreds of targets in response to multiple stresses [33]. Optimized expression of Hsf1p is important for yeast cells because either the deletion or overexpression of this gene leads to growth arrest [15]. To evaluate whether the lower expression activity of Hsf1p and related heat shock proteins was be beneficial or detrimental to YJS329 under stress conditions, we expressed the *HSF1* gene from BYZ1 in YJS329 using a low-copy plasmid. This genetic manipulation enhanced the cell viability of YJS329 by 57% and 25% after heat or ethanol treatment (*t* test, *P* < 0.05; Figure 5A), respectively, indicating that the appropriate readjustment of the expression of important transcription factors can contribute to the adaptability of yeast strains.

More glycerol might improve the taste of alcoholic beverages but is undesirable for bioethanol production. When *FPS1* (involved in efflux of glycerol; this gene showed lower expression in YJS329 compared with BYZ1) was deleted in YJS329 to produce the YJSΔ*FPS1* strain, the production of glycerol and acetic acid decreased and the conversion rate of glucose to ethanol improved by 1% compared with YJS329; however, the final concentration of ethanol was slightly less than in YJS329 because of the higher residual sugar in YJSΔ*FPS1* (*t* test, *P* <0.05; Figure 5B). Inspired by the different regulatory roles of *ALD6* in YJS329 and BYZ1, we explored the possibility to further reduce the production of glycerol in YJSΔ*FPS1* by overexpression of *ALD6*. Beyond our expectation, strain YJSΔ*FPS1ALD6* produced similar amounts of glycerol but 1.3% more ethanol (*t* test, *P* <0.05) than YJSΔ*FPS1* as a result of consuming more sugar than YJSΔ*FPS1*. We found that the over-expression of *ALD6* could enhance the tolerance of ethanol in both YJS329 and YJSΔ*FPS1* (*t* test, *P* <0.05; Figure 5C), which may explain the higher fermentation ability of strain YJSΔ*FPS1ALD6*. In addition, the over-expression of *ALD6* and deletion of *FPS1* significantly improved the tolerance of lignocellulosic hydrolysate (LH, contains inhibitors acetic acid, furan, and 5-HMF) in YJS329 (*t* test, *P* <0.05; Figure 5C), suggesting that this strategy may be useful for breeding industrial yeast strains with the ability to increase ethanol production from lignocellulosic biomass.

## Discussion

The genomic structural analysis (DNA content, PFGE, and aCGH analysis) indicated that YJS329 retained a diploid karyotype and had much lower structural polymorphisms than the bioethanol strain JAY270 and some other industrial strains [1,20]. We also sequenced the genome of YJSH2 (a haploid spore derived from the same tetrad as YJSH1) using the Illumina paired-ends method. After mapping the reads of YJSH2 to the YJSH1 genome, we estimated that the YJS329 genome had about 0.6 SNP/kb between allelic regions in homologous chromosomes (unpublished data). These results indicated that the YJS329 strain was genetically very stable, a desirable phenotype for industry practice. Although S288c has been widely used in scientific research, because of the high number of Ty elements, its genome seems to be more plastic [31,34]. High expression activity of Ty elements in genes was confirmed in the S288c-derived strain BYZ1 as a result of a dose effect (Additional file 4). The duplicated region on chromosome 4 in BYZ1 is probably the result of chromosomal translocations by ectopic recombination mediated by the flanking Ty elements. Strikingly, no dosage-compensation mechanisms acted to normalize the expression from each gene because the higher expression (1.59-fold) of this duplicated region almost matched the higher gene dose (1.5-fold). These results indicated that spontaneous Ty-driven rearrangements could be quite common and, if ignored, could easily lead to incorrect experimental results in genetic studies, especially for the S288c-derived strains.

Second-generation sequencing technology has proven to be an effective tool for the investigation of the genome sequences and structures of yeast strains and has provided many new insights into genome evolution and phenotypic effects [1,17,20,21,35,36]. The level of nucleotide polymorphisms between YJSH1 and S288c (0.57%) is very similar to the level separating S288c and AWRI1631 (wine strain), YJM789 (pathogenic strain), M22 (vineyard strain) or YPS163 (oak tree strain) [21,36], but, interestingly, YJSH1 was grouped closely with sake strains, consistent with their geographical distributions. To the best of our knowledge, YJS329 is the first bioethanol strain for which a high-quality assembled genome has been completed. The SNPs and indels that we have identified in the aligned regions of YJSH1 and S288c constitute the main genome mutations in these two strains. Mutation frequencies were found to be higher in the intergenic regions than in the coding regions, we found that up to 40% of the SNPs and 88% of the indels were located in intergenic sequences (accounting for about 27% of the genome). This pattern could arise from the sequence characteristics of intergenic regions (for example: the abundance of repeated sequences). However, we also observed a considerable number of mutations in the ORFs that play important roles in specified physiological activities. Remedying some of these mutations may improve the capabilities or change the specified phenotype of YJS329. A total of 11 ORFs were predicted in the YJS329 genome that are absent from the S288c genome. Remarkably, some of these ORFs may be very similar to those in other *Saccharomyces* species, including *S. paradoxus*, *S. carlsbergensis*, and *S. mikatae*. Therefore, during the evolution of the YJS329 genome, repeated yeast hybridization events that were followed by the gradual loss of one of the contributing genomes might have occurred. Undoubtedly, the genotypic characteristics of YJS329 that have been revealed in the present study will enrich the genetic resources of this species, which will be valuable for breeding strains with the desired phenotypes.

The recently developed RNA-Seq approach was used to explore the transcription profiles of the YJS329 and BYZ1 *S. cerevisiae* strains. Among the 2,611 differently expressed genes in these two strains, many were involved in the trehalose metabolism pathways, antioxidative factors, and membrane composition biosynthesis that are closely related to multiple stress-tolerance and fermentation characteristics. For example, consistent with the higher oleic acid content of membranes, the genes encoding the subunits of fatty acid synthetase (*FAS1* and *FAS2*), the acetyl-CoA carboxylase gene (*ACC1*), and the genes that function in fatty-acid desaturation and elongation (*ELO1* and *OLE1*) were considerably up-regulated in YJS329. Our results indicated that most of the differences in the physiological factors were consistent with the mRNA transcription differences between these two strains. Transcription –regulatory network analyses revealed that the transcription factors Msn2/4p, Hap1p, Hsf1p, and Arr1p might give prominence to the differently expressed genes and phenotypic differences between the two strains. This result was consistent with the observation that the *trans* variation is more common in expression polymorphism in yeast [37-39]. In spite of this, the contributions of *cis* variations on the divergence of mRNA expression and physiological metabolism should not be neglected because our results confirmed that mutations in the promoters of some important transcription factors and genes could directly affect the efficiency of their promoter efficiency. Overall, the molecular mechanisms underlying the mRNA expression differences between YJS329 and BYZ1 might involve: (i) SNPs and indels in the cis-acting elements that affect the expression efficiency of the genes; (ii) the inactivation of transcription factors by SNPs or indels; and (iii) changes in gene copy number. Remarkably, the discrepancies between the transcriptional profile (for example, of Hap1p) and the phenotype in the two strains might reflect variations in the activities of homologous proteins or posttranscriptional regulation, which deserve further assessment. In addition, here, for the first time, the expression activities of some novel ORFs under different conditions have been determined. Our study shows that whole-genome sequencing combined with RNA-Seq is a powerful tool for linking genotypes and phenotypes in functional genomic studies.

## Conclusions

A thorough understanding of the genetic variations and how these variations contribute to phenotypic diversities is vital for the development of excellent yeasts for industrial applications. In this study, functional genomics has revealed the genetic characteristics of a bioethanol strain YJS329 and compared it to the laboratory strain BYZ1. From the results of this study, targeted genetic strategies for YJS329 could be constructed. These strategies might include the introduction of wild type genes to remedy deleterious mutations in some of the strains, a heightening of the effects of beneficial mutations by gene deletion or overexpression, and the expression of novel genes to obtain specified functions. We expect that functional genomics studies of industrial microorganisms, such as those reported here, will, in the future, provide more effective means of improving breeding strategies to obtain the desired production traits.

## Methods

### Yeast strains and culture conditions

The S288c-isogenic strain BYZ1 (*MATa/MATα his3*Δ*1/his3*Δ*1 leu2*Δ*0/leu2*Δ*0 lys2*Δ*0/+ met15*Δ*0/+ ura3*Δ*0/ura3*Δ*0*) was generated from a cross between BY4741 and BY4742 (gift from Oliver Valerius, University of Göttingen, Germany). The yeast strain YJS329 (CCTCC 2011275) was isolated from a soil sample and was used for bioethanol production in Henan Tianguan Group Co., Ltd., China. Strain ZTW3 is a triploid strain that is stored in our laboratory. The growth medium (YPD) contained 10 g/L yeast extract, 20 g/L peptone, and 20 g/L glucose and had a pH of 5.5.

### Fermentation test

The fermentation medium contained 10/L yeast extract, 20 g/L peptone, and 160 or 280 g/L glucose. Yeast cells were precultured in YPD for 20 h at 30°C and transferred to the fermentation medium with an initial OD_600_ of 1. Three fermentation conditions were used: (i) 160 g/L glucose at 30°C; (ii) 160 g/L glucose at 40°C; and (iii) 280 g/L glucose at 30°C. Glucose and ethanol were measured as previously described [3].

### Analyses of physiological and biochemical factors

Yeast cells were cultured in 25 mL YPD with an initial OD_600_ of 0.05 and then collected at the early stationary phase (18 h, most genes involved in the stress response are induced at this phase). Trehalose, catalase, superoxide dismutase, and ergosterol were measured as previously described [3]. Glutathione was measured using a Glutathione Assay Kit according to the manufacturer's instructions (Nanjing Jiancheng Bioengineering Institute, China). Fatty acid was extracted by the method of Hama et al. [40] and then analyzed with a FOCUS GC Gas Chromatograph [41].

### PFGE and Array-comparative genomic hybridization

Yeast chromosomes were prepared as described by Argueso et al. [42] and separated by PFGE as described previously [41].

Total genomic DNA from BYZ1 and YJS329 was isolated with the yeast DNA kit (OMEGA, GA, USA) and then sonicated. The shearing DNA (200–1000 bp) was labeled with Cy5/Cy3 and hybridized to *S. cerevisiae* CGH 385 K Whole-Genome Tiling Arrays (NimbleGen). Scanning was performed with the Axon GenePix 4000B Microarray Scanner (Axon, USA). Raw data were extracted as pair files using NimbleScan software. Log2-ratio data were calculated and normalized by spatial correction and qspline fit normalization. DNA segments that contained three or more continuous probes with CNVs (|Log2-ratio| ≥0.35) were considered over- or under-represented regions. The microarray data have been deposited in the NCBI Gene Expression Omnibus [GEO:GSE31872].

### Whole genome sequencing and data analysis

Strain YJS329 was previously cultured in sporulation medium for 5 days, and an ascus with four ascospores was dissected to obtain four haploid strains (named YJSH1-4). YJSH1 was chosen for genome sequencing. Whole genome sequencing was performed on the 454 Life Sciences Genome Sequencer FLX (Roche) platform according to the manufacturer’s standard recommended sample preparation procedures. A shotgun sequencing library was constructed and a total of 718,904 reads were generated. 98.01% of the reads were assembled into 314 contigs using the Newbler software with the default parameters (minimum overlap length 40, minimum overlap identity 90%). The assembled sequences were manually checked, and some of the gaps were closed by Sanger sequencing reactions (contigs were first mapped to the corresponding chromosome and the sequences in gaps were amplified by PCR) to build the scaffolds. The 16 nuclear YJSH1 chromosomes were covered by 16 scaffolds including 30 contigs (Additional file 5). The sequences of the final contigs and scaffolds have been deposited with DDBJ/EMBL/GenBank under the Whole Genome Shotgun project [GenBank:AGAW00000000]. The version of the sequences described here is the first version of the sequences [GenBank:AGAW01000000].

SNPs were detected using the public BLASTN software [43] after the YJSH1 contig sequences were aligned to the individual S288c chromosome sequences [32]. The BLASTN parameters were adjusted as match = 4, mismatch = −5, gapopen = 3, gapextend = 5. Indels between the YJSH1 scaffolds and S288c chromosomes were detected using BLAT [44] (with default parameter) to reveal the physical gaps. The sizes and types (deletion or insertion) of indels were identified using the block sizes, qstarts, and tstarts information in the BLAT results file. Potential ORFs were predicted in two steps: (i) direct mapping of S288c ORFs from the *Saccharomyces* genome database by BLAT with the match length >95%, and (ii) using the Glimmer software (with the default parameters) to predict the ORFs located in unaligned regions of the YJSH1 contigs and S288c chromosomes [45]. The predicted ORFs were annotated by searching for their homologs in the NCBI non-redundant protein database. To predict structural variations, the YJSH1 scaffolds were aligned to the S288c chromosomes using the Artemis Comparative Tool [46]. The YJSH1 sequences that could not be aligned to the S288c genome were then compared against the contigs in the Whole Genome Shotgun database using BLASTN. Finally, PCRs were used to verify the predicted structural variations.

### RNA-Seq

The total RNA of each sample (three individual cultures of yeast cells) was extracted by the hot phenol method (growth conditions and time of extraction were identical to those used in the physiological factor analysis). cDNA libraries were prepared using the methods described by Pan and co-workers [47]. The cDNA library products were sequenced on the Illumina HiSeq™ 2000. The raw Illumina sequencing data have been deposited in NCBI’s GEO database [GEO:GSE31601]. After removing reads containing sequencing adapters and reads of low quality (reads in which the percentage of low quality bases (quality value ≤5) was more than 50%), the remaining clear reads were aligned to the *S. cerevisiae* S288c or YJSH1 genes with SOAPAligner [48]. The expression level was normalized by reads per kilobase of exon region per million mapped reads (RPKM) [49]. Screening of differentially expressed genes and *P*-value calculations were performed using the method proposed by Audic and Claverie [50]. The accuracy of the RNA-Seq experiment was verified by RT-qPCR.

### RT-qPCR

RNA extraction and quantitative PCR were performed as described by Tao et al. [41]. The primers that were used for quantitative PCR are listed in Additional file 15.

### Promoter efficiency evaluation

The promoters of *HSF1* (826 bp), *SFA1* (1250 bp), and *ALD6* (1199 bp) from BYZ1 were cloned into Sac I and Xho I sites before the Cre gene of plasmid pSH47 [GenBank:AF298782.1]. Inverse PCR was used to introduce the sequence mutations of YJS329 shown in Figure 4. The efficiency of the promoters was evaluated by the expression activity (RT-qPCR) of the report gene Cre. The values were represented by the log2 ratio of YJS329/BYZ1. The primers that were used for promoter cloning and RT-qPCR are listed in Additional file 15.

### Genetic manipulation

The full-length *HSF1* ORF along with 807 bp of the sequence upstream of the ORF was cloned into the CEN6 plasmid, pGFP-ble (derived from pGFP-N-FUS; the *URA3* marker was replaced by *ble*^*r*^). Deletion of the two copies of *FPS1* in YJS329 was performed as previously described [51]. In all cases, homozygous gene deletions were confirmed by diagnostic PCR. Overexpression of *ALD6* was carried out by cloning the *ALD6* ORF plus 1,005 bp of upstream sequence and 407 bp of downstream sequence into plasmid pYZ, which is derived from pYES2 (Invitrogen) but with *ble*^*r*^ replacing the *URA3* marker.

## Competing interests

The authors declare that they have no competing interests.

## Authors’ contributions

ZDQ and WXC designed the study and drafted the manuscript. ZDQ, WPM and LYD carried out the genome sequencing and molecular genetic studies. LTZ, LP, CJ and ZYH participated in the design of the study and performed the physiological and chemical analysis. All authors read and approved the final manuscript.

## Supplementary Material

Additional file 1 **Comparison of fermentation rates (CO**_2_**production) of YJS329 (cycle) and BYZ1 (triangle).** Fermentations were performed under (A) regular, (B) heat, and (C) high-gravity conditions motioned in the section of Material and Methods. Click here for file

Additional file 2 Comparison of the regions with copy number variations (CNVs) between YJS329 and BYZ1. Click here for file

Additional file 3 **Verification of the amplification of the DNA region of chromosome 4 in BY4742 genome.** Two pairs of primers (sequences were showed in Additional file 15) specified to genes *HMO1* and *UME6* were designed to verify the copy number variations of the ~60 kb region of chromosome 4 in BY4741, YJS329, and BY4742 genomes by RT-qPCR. Click here for file

Additional file 4 **Comparison of ethanol yield of YJS329 and YJSH1.** Fermentations were performed under regular, high gravity, and heat conditions described in the Methods. Click here for file

Additional file 5 Status and distribution of polymorphisms in each of the YJS329 chromosomes. Click here for file

Additional file 6 Details of the sequence variations detected in the YJSH1 genome. Click here for file

Additional file 7 Details of the YJSH1gene annotations. Click here for file

Additional file 8 RNA-seq reads mapping to S288c genome and genes. Click here for file

Additional file 9 Differentially expressed genes of YJS329 and BYZ1 revealed by RNA-Seq. Click here for file

Additional file 10 **Functional classification and transcriptional-regulation analysis of genes expressed differently in BYZ1 and YJS329.** (A) GO functional enrichment analysis of genes expressed differently in BYZ1 and YJS329 (FDR < 0.05). Orange pillars represent the classification of up-regulated genes in YJS329, and olive pillars represent the down-regulated genes. (B) Regulation network analysis of some key trans-transcriptional factors and their target genes. These genes were grouped into five terms marked with different color borders, including trehalose metabolism (black), antioxidative factors (green), heat-shock proteins (red), and fatty-acid and ergosterol metabolism (blue). Regulation relationships are presented by the arrows linking the nodes. The genes up-regulated or down-regulated genes with respect to BYZ1 are shown in red and green, respectively, and the color gradient represents the extent of regulation. Click here for file

Additional file 11 Comparison of the expression levels of stress-related genes between BYZ1 and YJS329. Click here for file

Additional file 12 **The different efficiencies of the promoters of *HSF1, SFA1 *, and *ALD6 *between BYZ1 and YJS329.** The efficiency of the promoters was evaluated by the expression activity of report gene Cre. The values were represented by log2 ratio of YJS329/BYZ1. Error bars represent SD of three independent samples. Format: DOC. Click here for file

Additional file 13 **The effects of *ALD6 *deletion on metabolites yield of ethanol fermentation. ** Yeast cells were precultured in YPD overnight, and were then transferred to the fermentation medium (10/L yeast extract, 20 g/L peptone, and 160 g/L glucose) with the initial OD_600_ of 1. Fermentations were performed at 30°C for 55 h.) Click here for file

Additional file 14 **Verification of the transcription of some novel genes.** (A) The expression level and boundary of the novel ORF chr06.003. (B) Relative expression of five novel ORFs under different conditions. YJS329 was grown in YPD medium with initial OD600 of 0.05, and total RNA were then extracted at 7 h (exponential phase), 15 h (diauxic growth), and 25 h (stationary) for determination of the expression of these novel genes. "Fermentation" indicates the total RNA extracted at 20 h under ethanol-fermentation conditions (33°C) with corn mash as the feedstock (containing 270 g/L glucose.) Click here for file

Additional file 15 Primers used in this study. Click here for file
