# Supplementary material for: Genome sequencing and genetic breeding of a bioethanol Saccharomyces cerevisiae strain YJS329
Source: BMC Genomics. 2012 Sep 15;13:479. doi: 10.1186/1471-2164-13-479 (PMC3484046; doi:10.1186/1471-2164-13-479)
Supplement: Additional file 1 — Comparison of fermentation rates (CO2production) of YJS329 (cycle) and BYZ1 (triangle). Fermentations were performed under (A) regular, (B) heat, and (C) high-gravity conditions motioned in the section of Material and Methods. [file 1471-2164-13-479-S1.doc]

**
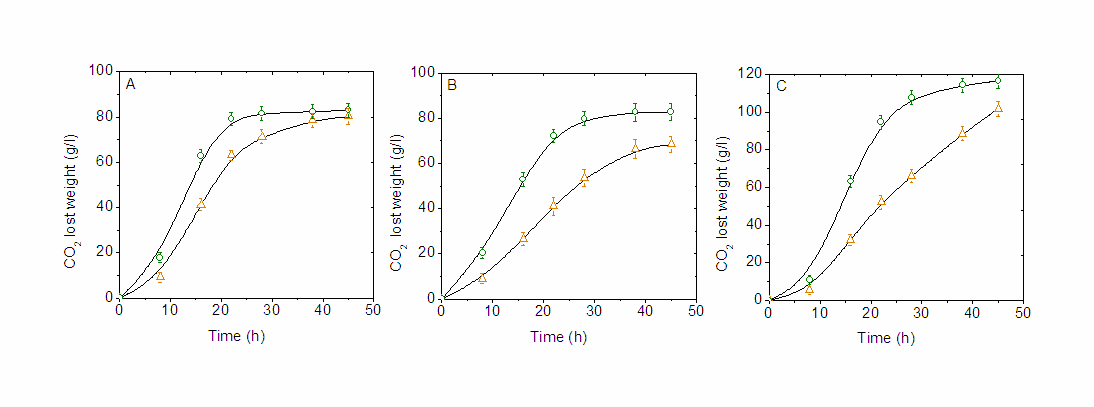
**

**Additional file 1**. Comparison of fermentation rates (CO_2_ production) of YJS329 (cycle) and BYZ1 (triangle). Fermentations were performed under (A) regular, (B) heat, and (C) high-gravity conditions motioned in the section of Material and Methods.
