# Supplementary material for: Genome sequencing and genetic breeding of a bioethanol Saccharomyces cerevisiae strain YJS329
Source: BMC Genomics. 2012 Sep 15;13:479. doi: 10.1186/1471-2164-13-479 (PMC3484046; doi:10.1186/1471-2164-13-479)
Supplement: Additional file 3 — Verification of the amplification of the DNA region of chromosome 4 in BY4742 genome. Two pairs of primers (sequences were showed in Additional file 15) specified to genes HMO1 and UME6 were designed to verify the copy number variations of the ~60 kb region of chromosome 4 in BY4741, YJS329, and BY4742 genomes by RT-qPCR. [file 1471-2164-13-479-S3.doc]

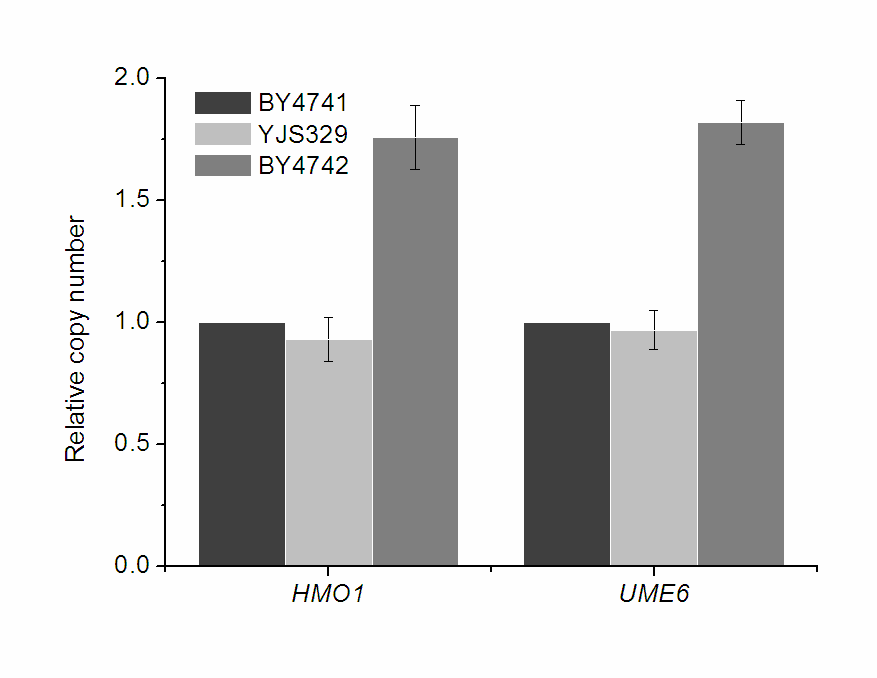


**Additional file 3.** Verification of the amplification of the DNA region of chromosome 4 in BY4742 genome. Two pairs of primers (sequences were showed in Additional file 15) specified to genes *HMO1* and *UME6* were designed to verify the copy number variations of the ~60kb region of chromosome 4 in BY4741, YJS329, and BY4742 genomes by RT-qPCR.
