# Supplementary material for: Genome sequencing and genetic breeding of a bioethanol Saccharomyces cerevisiae strain YJS329
Source: BMC Genomics. 2012 Sep 15;13:479. doi: 10.1186/1471-2164-13-479 (PMC3484046; doi:10.1186/1471-2164-13-479)
Supplement: Additional file 4 — Comparison of ethanol yield of YJS329 and YJSH1. Fermentations were performed under regular, high gravity, and heat conditions described in the Methods. [file 1471-2164-13-479-S4.doc]

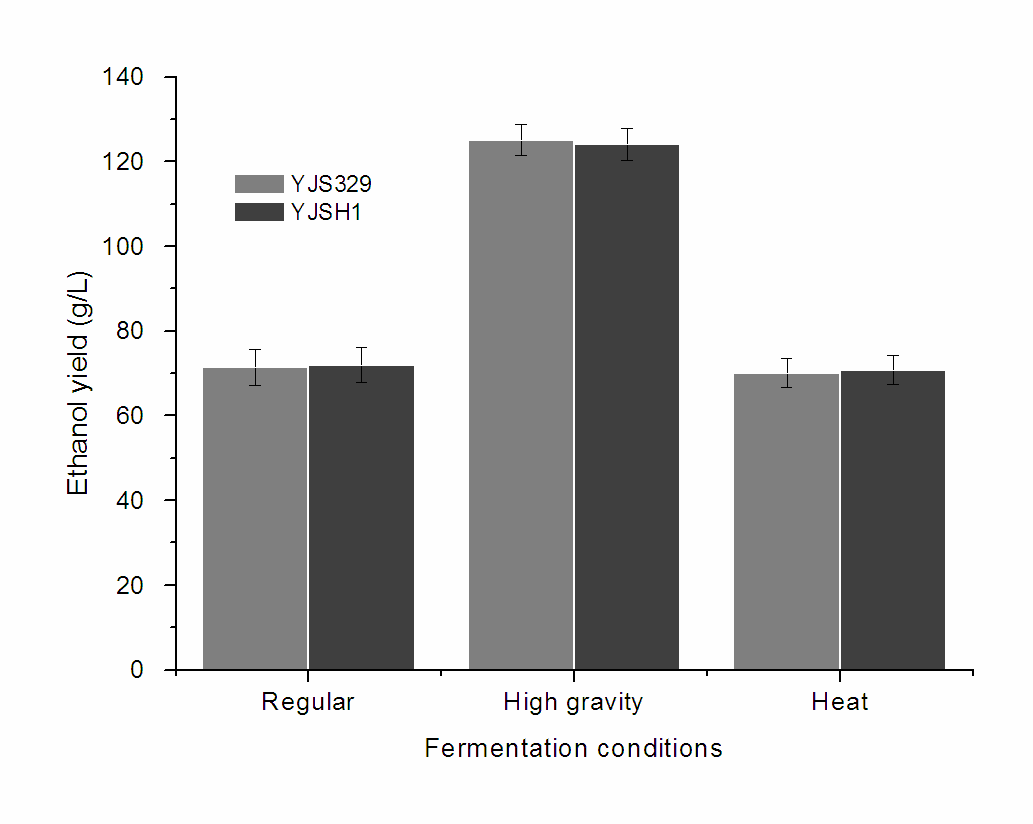


**Additional file 4.** Comparison of ethanol yield of YJS329 and YJSH1. Fermentations were performed under regular, high gravity, and heat conditions described in the Methods.
