# Supplementary material for: Genome sequencing and genetic breeding of a bioethanol Saccharomyces cerevisiae strain YJS329
Source: BMC Genomics. 2012 Sep 15;13:479. doi: 10.1186/1471-2164-13-479 (PMC3484046; doi:10.1186/1471-2164-13-479)
Supplement: Additional file 5 — Status and distribution of polymorphisms in each of the YJS329 chromosomes. [file 1471-2164-13-479-S5.doc]

**Additional file 5.** Status and distribution of polymorphisms in each of the YJS329 chromosomes.

| Chr  number | Contigs | SNP | SNP/kb | Insert | Delete | Indels/kb |
| --- | --- | --- | --- | --- | --- | --- |
| 1 | 3 | 1739 | 9.62 | 104 | 116 | 1.22 |
| 2 | 1 | 3073 | 3.97 | 158 | 198 | 0.46 |
| 3 | 1 | 2087 | 7.11 | 149 | 148 | 1.01 |
| 4 | 2 | 10154 | 6.96 | 453 | 498 | 0.65 |
| 5 | 3 | 3268 | 5.96 | 154 | 193 | 0.63 |
| 6 | 1 | 1303 | 5.25 | 85 | 105 | 0.77 |
| 7 | 2 | 6341 | 6.19 | 337 | 342 | 0.66 |
| 8 | 1 | 3065 | 6.13 | 144 | 174 | 0.64 |
| 9 | 2 | 2346 | 5.85 | 143 | 133 | 0.69 |
| 10 | 1 | 3276 | 4.69 | 166 | 198 | 0.52 |
| 11 | 3 | 3054 | 4.76 | 211 | 175 | 0.60 |
| 12 | 3 | 4870 | 4.86 | 265 | 303 | 0.57 |
| 13 | 3 | 4650 | 5.21 | 234 | 264 | 0.56 |
| 14 | 1 | 4729 | 6.31 | 241 | 257 | 0.66 |
| 15 | 2 | 5434 | 5.26 | 269 | 323 | 0.57 |
| 16 | 1 | 5609 | 9.62 | 266 | 287 | 0.62 |
