# Supplementary material for: Genome sequencing and genetic breeding of a bioethanol Saccharomyces cerevisiae strain YJS329
Source: BMC Genomics. 2012 Sep 15;13:479. doi: 10.1186/1471-2164-13-479 (PMC3484046; doi:10.1186/1471-2164-13-479)
Supplement: Additional file 8 — RNA-seq reads mapping to S288c genome and genes. [file 1471-2164-13-479-S8.doc]

**Additional file 8** RNA-seq reads mapping to S288c genome and genes.

|  | Map to genome | |  | Map to gene | |
| --- | --- | --- | --- | --- | --- |
|  | BYZ1 | YJS329 |  | BYZ1 | YJS329 |
|  | reads number | reads number |  | reads number | reads number |
| Total Reads | 5812819 | 5868877 |  | 5812819 | 5868877 |
| Total Base Pairs | 284828131 | 287574973 |  | 284828131 | 287574973 |
| Total Mapped Reads | 5691370 | 5689582 |  | 4973917 | 4992813 |
| Perfect match | 5272140 | 4393732 |  | 4611578 | 3871478 |
| Mismatch | 419230 | 1295850 |  | 362339 | 1121335 |
| Unique match | 5262880 | 5359452 |  | 4687047 | 4772290 |
| Multi-position match | 428490 | 330130 |  | 286870 | 220523 |
| Total Unmapped Reads | 121449 | 179295 |  | 838902 | 876064 |
