# Supplementary material for: Genome sequencing and genetic breeding of a bioethanol Saccharomyces cerevisiae strain YJS329
Source: BMC Genomics. 2012 Sep 15;13:479. doi: 10.1186/1471-2164-13-479 (PMC3484046; doi:10.1186/1471-2164-13-479)
Supplement: Additional file 10 — Functional classification and transcriptional-regulation analysis of genes expressed differently in BYZ1 and YJS329. (A) GO functional enrichment analysis of genes expressed differently in BYZ1 and YJS329 (FDR < 0.05). Orange pillars represent the classification of up-regulated genes in YJS329, and olive pillars represent the down-regulated genes. (B) Regulation network analysis of some key trans-transcriptional factors and their target genes. These genes were grouped into five terms marked with different color borders, including trehalose metabolism (black), antioxidative factors (green), heat-shock proteins (red), and fatty-acid and ergosterol metabolism (blue). Regulation relationships are presented by the arrows linking the nodes. The genes up-regulated or down-regulated genes with respect to BYZ1 are shown in red and green, respectively, and the color gradient represents the extent of regulation. [file 1471-2164-13-479-S10.doc]

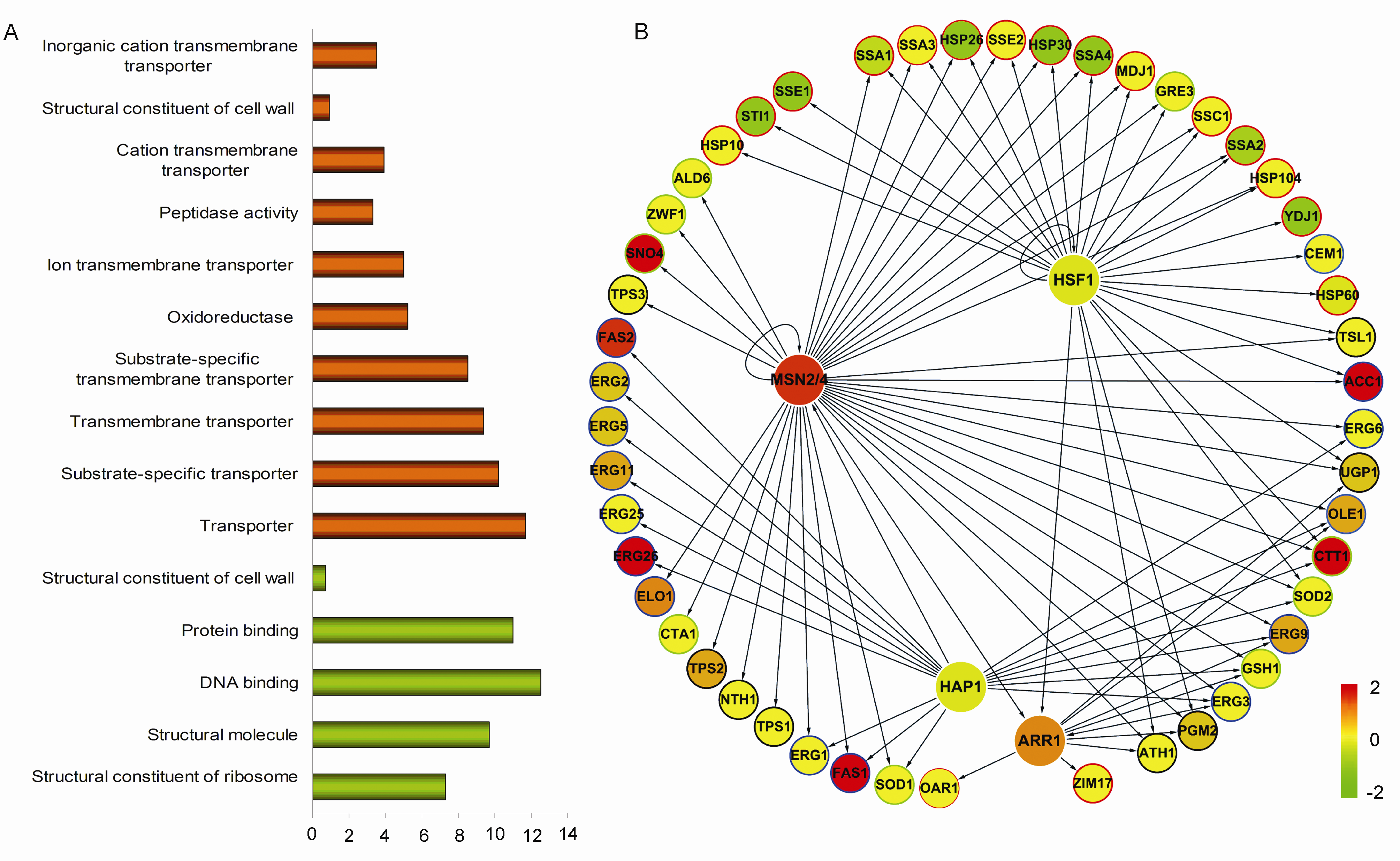


**Additional file 10.** Functional classification and transcriptional-regulation analysis of genes expressed differently in BYZ1 and YJS329. (A) GO functional enrichment analysis of genes expressed differently in BYZ1 and YJS329 (FDR < 0.05). Orange pillars represent the classification of up-regulated genes in YJS329, and olive pillars represent the down-regulated genes. (B) Regulation network analysis of some key trans-transcriptional factors and their target genes. These genes were grouped into five terms marked with different color borders, including trehalose metabolism (black), antioxidative factors (green), heat-shock proteins (red), and fatty-acid and ergosterol metabolism (blue). Regulation relationships are presented by the arrows linking the nodes. The genes up-regulated or down-regulated genes with respect to BYZ1 are shown in red and green, respectively, and the color gradient represents the extent of regulation.
