# Supplementary material for: Genome sequencing and genetic breeding of a bioethanol Saccharomyces cerevisiae strain YJS329
Source: BMC Genomics. 2012 Sep 15;13:479. doi: 10.1186/1471-2164-13-479 (PMC3484046; doi:10.1186/1471-2164-13-479)
Supplement: Additional file 11 — Comparison of the expression levels of stress-related genes between BYZ1 and YJS329. [file 1471-2164-13-479-S11.doc]

**Additional file 11.** Comparison of the expression levels of stress-related genes between BYZ1 and YJS329.

| Category | Genes | log2 Ratio(YJS329/BYZ1) | Up or Down | *P*-value |
| --- | --- | --- | --- | --- |
| Transcription factors | *HSF1* | -0.61 | Down | 4.77E-22 |
|  | *MSN4* | 1.38 | Up | 6.07E-13 |
|  | *MSN2* | 0.78 | Up | 0 |
|  | *HAP1* | -0.66 | Down | 8.24E-85 |
|  | *ARR1* | 1.02 | Up | 3.63E-13 |
|  |  |  |  |  |
| Genes in ergosterol synthesis | *ERG9* | 0.89 | Up | 4.22E-12 |
|  | *ERG24* | 0.86 | Up | 6.06E-13 |
|  | *ERG11* | 0.80 | Up | 0 |
|  | *ERG10* | 0.73 | Up | 3.63E-12 |
|  | *ERG2* | 0.69 | Up | 1.15E-12 |
|  | *ERG8* | 0.57 | Up | 2.77E-06 |
|  | *ERG1* | 0.55 | Up | 0 |
|  | *ERG25* | 0.41 | Up | 0 |
|  | *ERG27* | 0.38 | Up | 5.83E-05 |
|  | *ERG12* | 0.37 | Up | 0.00014 |
|  | *ERG3* | 0.28 | Up | 0 |
|  | *ERG6* | -0.58 | Down | 1.34E-21 |
|  | *ERG20* | -0.43 | Down | 3.39E-17 |
|  |  |  |  |  |
| Genes in fatty acid synthesis | *FAS2* | 1.49 | Up | 2.4E-11 |
|  | *FAS1* | 2.25 | Up | 0 |
|  | *ELO1* | 1.02 | Up | 6.85E-12 |
|  | *OLE1* | 0.90 | Up | 0 |
|  |  |  |  |  |
| Catalase | *CTT1* | 3.23 | Up | 1.85E-11 |
|  | *CTA1* | 0.24 | Up | 2.49E-11 |
|  |  |  |  |  |
| Genes in trehalose synthesis | *TPS2* | 0.88 | Up | 0 |
|  | *TSL1* | 0.30 | Up | 2.63E-11 |
|  | *NTH2* | 0.22 | Up | 0.000194 |
|  | *TPS1* | -0.15 | Down | 3.74E-08 |
