# Supplementary material for: Genome sequencing and genetic breeding of a bioethanol Saccharomyces cerevisiae strain YJS329
Source: BMC Genomics. 2012 Sep 15;13:479. doi: 10.1186/1471-2164-13-479 (PMC3484046; doi:10.1186/1471-2164-13-479)
Supplement: Additional file 12 — The different efficiencies of the promoters of HSF1, SFA1 , and ALD6 between BYZ1 and YJS329. The efficiency of the promoters was evaluated by the expression activity of report gene Cre. The values were represented by log2 ratio of YJS329/BYZ1. Error bars represent SD of three independent samples. Format: DOC. [file 1471-2164-13-479-S12.doc]

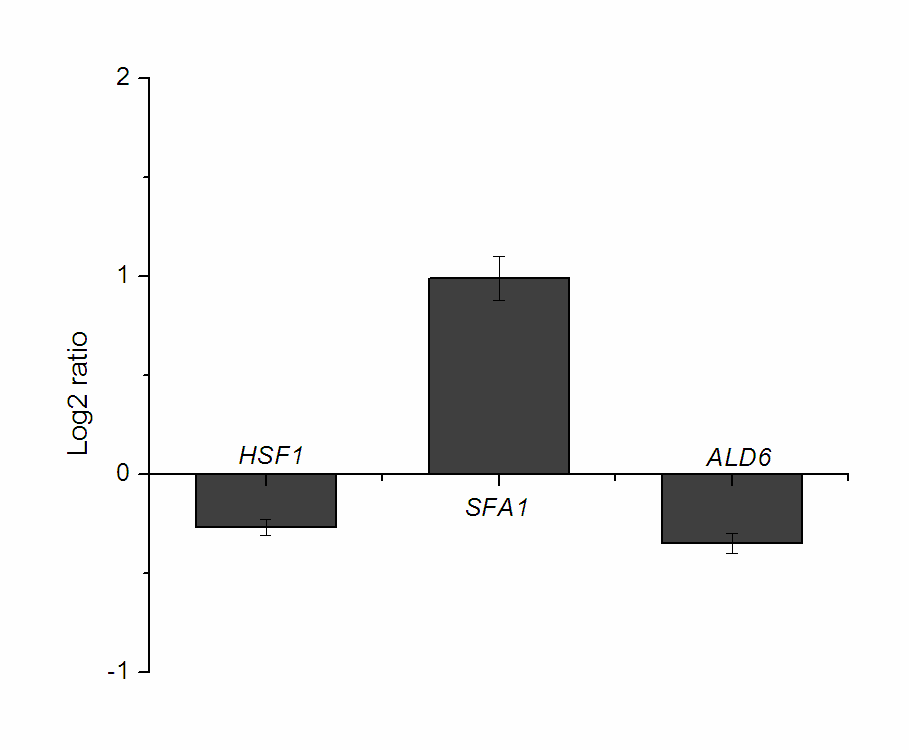


**Additional file 12.** The different efficiencies of the promoters of *HSF1*, *SFA1*, and *ALD6* between BYZ1 and YJS329. The efficiency of the promoters was evaluated by the expression activity of report gene Cre. The values were represented by log2 ratio of YJS329/BYZ1. Error bars represent SD of three independent samples.
