# Supplementary material for: Genome sequencing and genetic breeding of a bioethanol Saccharomyces cerevisiae strain YJS329
Source: BMC Genomics. 2012 Sep 15;13:479. doi: 10.1186/1471-2164-13-479 (PMC3484046; doi:10.1186/1471-2164-13-479)
Supplement: Additional file 13 — The effects of ALD6 deletion on metabolites yield of ethanol fermentation. Yeast cells were precultured in YPD overnight, and were then transferred to the fermentation medium (10/L yeast extract, 20 g/L peptone, and 160 g/L glucose) with the initial OD600 of 1. Fermentations were performed at 30°C for 55 h.) [file 1471-2164-13-479-S13.doc]

**
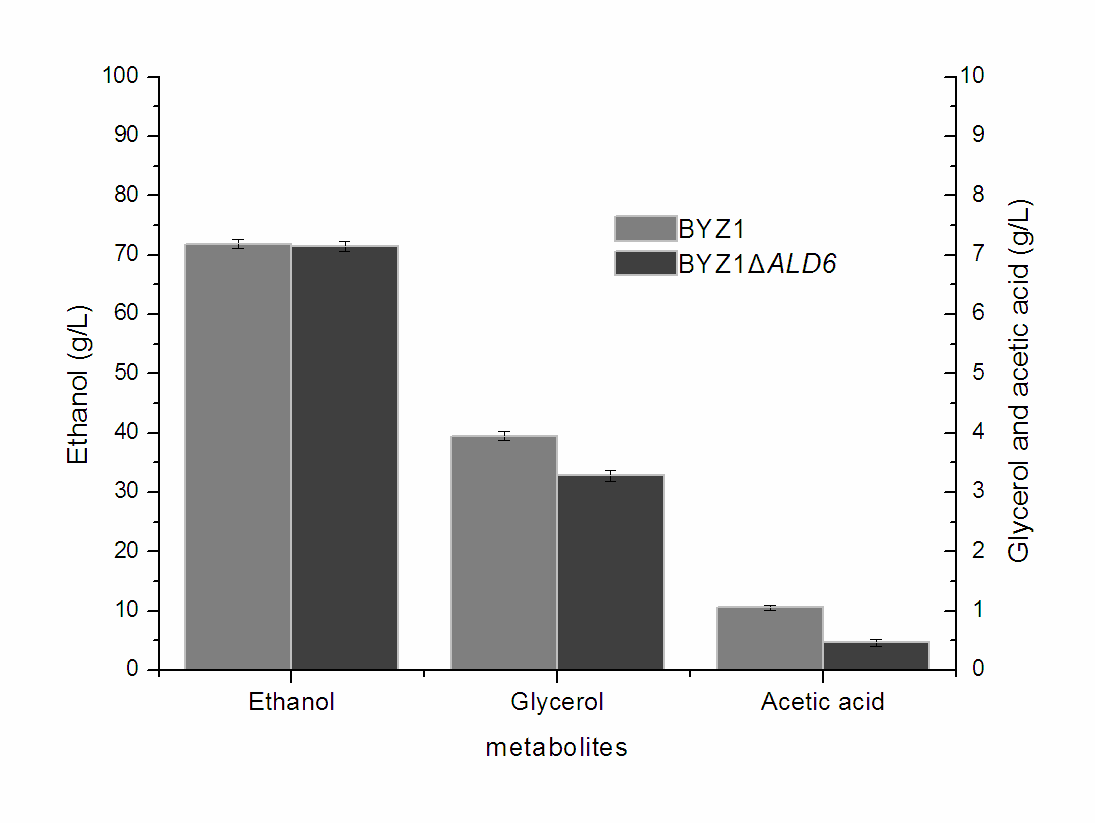
**

**Additional file 13.** The effects of *ALD6* deletion on metabolites yield of ethanol fermentation. Yeast cells were precultured in YPD overnight, and were then transferred to the fermentation medium (10 /L yeast extract, 20g/L peptone, and 160 g/L glucose) with the initial OD_600_ of 1. Fermentations were performed at 30 °C for 55 h.
