# Supplementary material for: Genome sequencing and genetic breeding of a bioethanol Saccharomyces cerevisiae strain YJS329
Source: BMC Genomics. 2012 Sep 15;13:479. doi: 10.1186/1471-2164-13-479 (PMC3484046; doi:10.1186/1471-2164-13-479)
Supplement: Additional file 14 — Verification of the transcription of some novel genes. (A) The expression level and boundary of the novel ORF chr06.003. (B) Relative expression of five novel ORFs under different conditions. YJS329 was grown in YPD medium with initial OD600 of 0.05, and total RNA were then extracted at 7 h (exponential phase), 15 h (diauxic growth), and 25 h (stationary) for determination of the expression of these novel genes. "Fermentation" indicates the total RNA extracted at 20 h under ethanol-fermentation conditions (33°C) with corn mash as the feedstock (containing 270 g/L glucose.) [file 1471-2164-13-479-S14.doc]

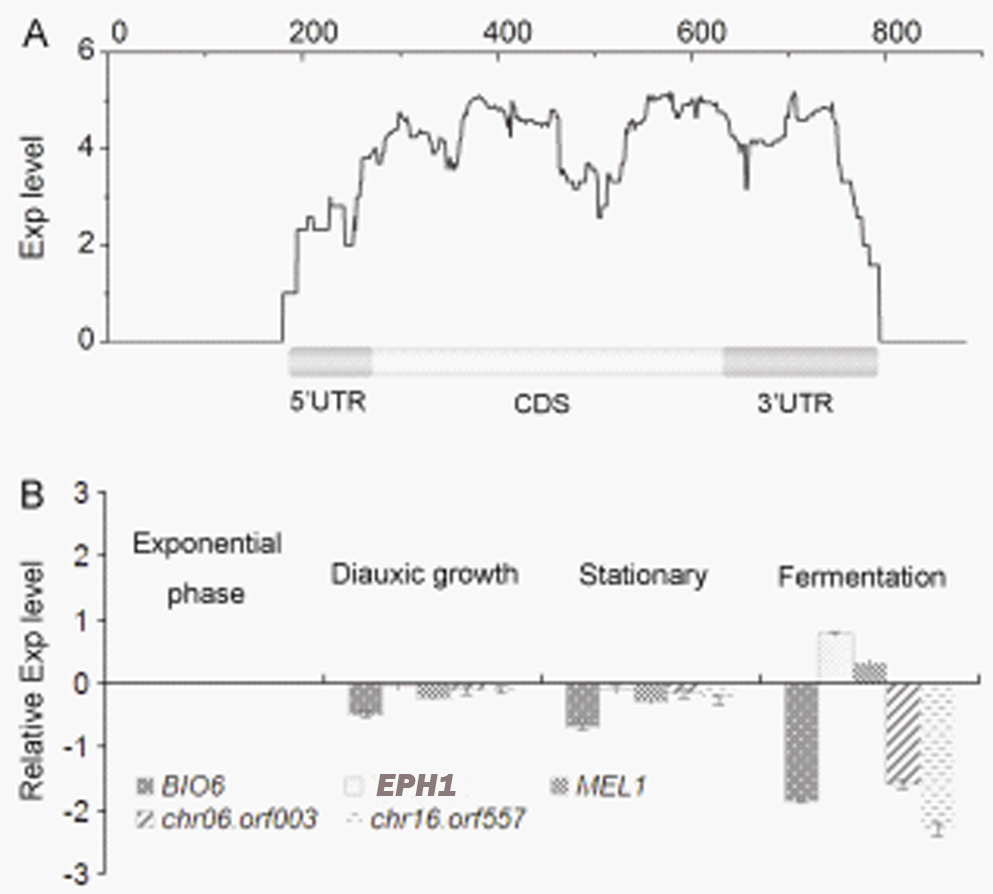


**Additional file 14.** Verification of the transcription of some novel genes. (A) The expression level and boundary of the novel ORF chr06.003. (B) Relative expression of five novel ORFs under different conditions. YJS329 was grown in YPD medium with initial OD600 of 0.05, and total RNA were then extracted at 7 hours (exponential phase), 15 hours (diauxic growth), and 25 hours (stationary) for determination of the expression of these novel genes. ‘Fermentation’ indicates the total RNA extracted at 20 hours under ethanol-fermentation conditions (33°C) with corn mash as the feedstock (containing 270 g/L glucose).
