# Supplementary material for: Genome sequencing and genetic breeding of a bioethanol Saccharomyces cerevisiae strain YJS329
Source: BMC Genomics. 2012 Sep 15;13:479. doi: 10.1186/1471-2164-13-479 (PMC3484046; doi:10.1186/1471-2164-13-479)
Supplement: Additional file 15 — Primers used in this study. [file 1471-2164-13-479-S15.doc]

**Additional file 15.** Primers used in this study.

| Primer number^a^ | Name | Sequence 5'-3' | size (bp) | Efficiency^b^ |
| --- | --- | --- | --- | --- |
| 1 | ARR1-F | GGACTGAAATAAAGGAGCC | 103 | 0.95 |
|  | ARR1-R | TGTCTTGGATGGAAACCC |  |  |
| 2 | HMO1-F | AAGATGCTATTATCGCTGCTC | 379 | 1.02 |
|  | HMO1-R | GCGTGCTGTAAACTGAAAGG |  |  |
| 3 | UME6-F | TGAGTCAAGGACAAGGCAAGTT | 257 | 0.99 |
|  | UME6-R | CGTTGGGATAATCAGCGGTA |  |  |
| 4 | TPS1-F | AGGCTGGATTACATCAAAGG | 293 | 0.93 |
|  | TPS1-R | GTGGACGAGACCAAACAAAC |  |  |
| 5 | TPS2-F | ACGCCAAAGAACTGAAAGAA | 228 | 0.98 |
|  | TPS2-R | CATCACCCAAACATAATACA |  |  |
| 6 | TPS3-F | TAAACATCGGATTGATAGCG | 237 | 0.99 |
|  | TPS3-R | TTATGTGCGTAATGGCTTCC |  |  |
| 7 | MSN4-F | GTCGGATAATGCCTCTGC | 196 | 1.07 |
|  | MSN4-R | GTTTGGGCTTACCGTTGC |  |  |
| 8 | HSF1-F | ACAAGTGTCAATCAAGGCGAA | 192 | 1.02 |
|  | HSF1-R | CTGGGTGGAATAACTGTGGTC |  |  |
| 9 | SFA1-F | AAAGGTTGGGGTCAATCTATTATCAT | 279 | 0.98 |
|  | SFA1-R | TTCATCAGACTTCAAGACGGTTC |  |  |
| 10 | ALD6-F | GTGCTATCACTAACCGTCA | 400 | 0.96 |
|  | ALD6-R | CTACCGTAACCAGATTGCT |  |  |
| 11 | BIO6-F | AGTCCGCTTTGATTGTCGT | 295 | 1.05 |
|  | BIO6-R | GACTATGTCTGGCACTATCCCT |  |  |
| 12 | YJS-HE-F | GAAGCAAGTCGCAACCAGTC | 390 | 0.99 |
|  | YJS-HE-R | ACTGCCACAACTATGTATCTT |  |  |
| 13 | chr02.orf010-F | TGGTGCCAATGTGAATAACT | 254 | 1.08 |
|  | chr02.orf010-R | AAATCTCTTCCAAGGTCGTG |  |  |
| 14 | chr06.orf003-F | CTGGTGCTGTTGTTTCCTCT | 176 | 0.98 |
|  | chr06.orf003-R | AACCTCTTCATTTTTCATTCTCC |  |  |
| 15 | chr06.orf127-F | GGCTGGTAATAAGGGTGCT | 259 | 0.97 |
|  | chr06.orf128-R | GAAACCTGCCTCTATGAAAC |  |  |
| 16 | ACT1-F | GGCTTCTTTGACTACCTTCCA | 89 | 1.09 |
|  | ACT1-R | AGAAACACTTGTGGTGAACGA |  |  |
| 17 | pHSF1-F | GGCAATCGAAGCTCATAG | 504 |  |
|  | pHSF1-R | TTGCCTTAGGTTACCATTAC |  |  |
| 18 | pSFA1-F | AAGTTTCTCCTTATCCTCCAT | 904 |  |
|  | pSFA1-R | GTAGCGGCGGACATTCT |  |  |
| 19 | pALD6-F | AACCGTACTCACAACTTTCCG | 1211 |  |
|  | pALD6-R | GCATAGGCAGCAGCATCTC |  |  |
| 20 | Ho-F | AAACACGACTATTCTGATGGCTA | 561 |  |
|  | Ho-R | AACACCATTTCCTGCGAGTA |  |  |

**Table S3** Primers used in this study

| Primer number^a^ | Name | Sequence 5'-3' | Amplicon size (bp) | Efficiency^b^ |
| --- | --- | --- | --- | --- |
| 21 | pcALD6-F | TCAU*GAGCTC*UTACGTATCTATGTGTGTA | 1199 |  |
|  | pcALD6R | TCU*GAATTC*UTGATAGTATGTGTTTGTG |  |  |
| 22 | pcSFA1-F | CCAU*GAGCTC*UACTTGTATTCTTTGGTAGT | 1250 |  |
|  | pcSFA1R | GCGGU*GAATTC*UTACTTATATTACTTATATTCCTT |  |  |
| 23 | pcHSF1F | TCAU*GAGCTC*UCCACTTTTCTGTGCCTTTT | 826 |  |
|  | pcHSF1-R | TAU*GAATTC*UGCGCCAACAATACAGCTGTC |  |  |
| 24 | Cre-F | CCAGGTATTGTTAGCGGT | 291 | 1.01 |
|  | Cre-R | CACATCATCCACGGTTCT |  |  |
| 25 | OHSF1-F | GAU*GAGCTC*UCCACTTTTCTGTGCCTTTTG | 3357 |  |
|  | OHSF1-R | CGU*GAATTC*UTACGCTATTTAATGACCTTGCC |  |  |
| 26 | OALD6-F | TCU*GAGCTC*UCCTAATGATCTGATGCGCT | 2935 |  |
|  | OALD6-R | TTGCU*CTCGAG*UTTCGCAGTGTTTATGTTATA |  |  |

^a^ Primers 1–10 were used to verify the mRNA expression level of the relevant genes in the RNA-seq experiment by RT-qPCR; primers 1–3 were also used to verify the copy number variation of relevant DNA regions by RT-qPCR; primers 11-15 were used to detect the expression of relevant novel genes; primer 16 was used as the internal reference of RT-qPCR; primers 17–20 were used in PCR to confirm the sequence mutations of selected genes; primers 21–23 were used to clone the promoters of genes *ALD6*, *SFA1*, and *HSF1*; primer 24 was designed to evaluate the efficiency of promoters cloned into the plasmid pSH47; and primers 25 and 26 were used to clone *HSF1* and *ALD6*, respectively.

^b^ Efficiency (*E*) was determined using the formula *E*=10^(-1/slope)^-1,with the “slope” being the slope of the standard curve that was obtained from 10 fold serial dilution of template.
